# The CIREL Cohort: A Prospective Controlled Registry Studying the Real-Life Use of Irinotecan-Loaded Chemoembolisation in Colorectal Cancer Liver Metastases: Interim Analysis

**DOI:** 10.1007/s00270-020-02646-8

**Published:** 2020-09-24

**Authors:** Philippe L. Pereira, Roberto Iezzi, Riccardo Manfredi, Francesca Carchesio, Zoltan Bánsághi, Elias Brountzos, Stavros Spiliopoulos, Javier J. Echevarria-Uraga, Belarmino Gonçalves, Riccardo Inchingolo, Michele Nardella, Olivier Pellerin, Maria Sousa, Dirk Arnold, Thierry de Baère, Fernando Gomez, Thomas Helmberger, Geert Maleux, Hans Prenen, Bruno Sangro, Bleranda Zeka, Nathalie Kaufmann, Julien Taieb

**Affiliations:** 1 Zentrum für Radiologie, Minimal-Invasive Therapien und Nuklearmedizin, SLK-Kliniken Heilbronn GmbH, Am Gesundbrunnen 20-26, 74078 Heilbronn, Germany; 2 Dipartimento di Diagnostica per Immagini, Radioterapia Oncologica ed Ematologia, UOC di Radiologia Diagnostica ed Interventistica Generale, Fondazione Policlinico Universitario “A. Gemelli” IRCCS, Rome, Italy; 3 Medical Imaging Center, Semmelweis University, Korányi Sándor u. 2, Budapest, 1082 Hungary; 4 Interventional Radiology Unit, 2nd Department of Radiology, School of Medicine, National and Kapodistrian University of Athens, Attikon University General Hospital, Rimini 1, Chaidari, 124 62 Athens, Greece; 5 Department of Radiology, Osakidetza Basque Health Service, Galdakao-Usansolo Hospital, Barrio Labeaga s/n, 48960 Galdakao, Spain; 6 Department of Interventional Radiology, Portuguese Oncology Institute, Rua Dr. António Bernardino de Almeida, 4200-072 Porto, Portugal; 7 Division of Interventional Radiology, Department of Radiology, Madonna delle Grazie Hospital, Via Montescaglioso, 75100 Matera, Italy; 8 Assistance Publique Hôpitaux de Paris, Service de Radiologie Interventionnelle Vasculaire et Oncologique, Hôpital Européen Georges Pompidou, Université Paris Descartes, Sorbonne Paris-Cité, 20 Rue Leblanc, 75015 Paris, France; 9 Asklepios Tumorzentrum Hamburg, AK Altona, Paul Ehrlich Str. 1, 22763 Hamburg, Germany; 10 Service de Radiologie Interventionelle, Institut Gustave Roussy, 114 Rue Edouard Vaillant, 94800 Villejuif, France; 11 Servicio de Radiodiagnóstico, Hospital Clínic de Barcelona, Calle Villarroel, 170, 08036 Barcelona, Spain; 12 Department of Radiology, The Netherlands Cancer Institute, Plesmanlaan 121, 1066 CX Amsterdam, The Netherlands; 13 Institut für Radiologie, München Klinik Bogenhausen Neuroradiologie und minimal-invasive Therapie, Englschalkinger Str. 77, 81925 Munich, Germany; 14 Radiologie, UZ Leuven, Herestraat 49, 3000 Louvain, Belgium; 15 Oncology Department, UZ Antwerp, Wilrijkstraat 10, 2650 Edegem, Belgium; 16 Liver Unit, Clinica Universidad de Navarra-IDISNA and CIBEREHD, Av. de Pío XII 36, 31008 Pamplona, Spain; 17 Clinical Research Department, Cardiovascular and Interventional Radiological Society of Europe, Neutorgasse 9, 1010 Vienna, Austria; 18 Assistance Publique Hôpitaux de Paris, Service d’hepatogastroentérologie et d’oncologie digestive, Hôpital Européen Georges Pompidou, Université Paris Descartes, Sorbonne Paris-Cité, 20 Rue Leblanc, 75015 Paris, France

**Keywords:** TACE, Drug-eluting microspheres, Irinotecan, Chemoembolisation, Interim analysis

## Abstract

**Purpose:**

Transarterial chemoembolisation (TACE) using irinotecan-eluting beads is an additional treatment option for colorectal cancer liver metastases (CRLM) patients that are not eligible for curative treatment approaches. This interim analysis focuses on feasibility of the planned statistical analysis regarding data distribution and completeness, treatment intention, safety and health-related quality of life (HRQOL) of the first 50 patients prospectively enrolled in the CIrse REgistry for LifePearl™ microspheres (CIREL), an observational multicentre study conducted across Europe.

**Methods:**

In total, 50 patients ≥ 18 years diagnosed with CRLM and decided to be treated with irinotecan-eluting LifePearl™ microspheres TACE (LP-irinotecan TACE) by a multidisciplinary tumour board. There were no further inclusion or exclusion criteria. The primary endpoint is the categorisation of treatment intention, and secondary endpoints presented in this interim analysis are safety, treatment considerations and HRQOL.

**Results:**

LP-irinotecan TACE was conducted in 42% of patients as salvage therapy, 20% as an intensification treatment, 16% as a first-line treatment, 14% a consolidation treatment and 8% combination treatment with ablation with curative intent. Grade 3 and 4 adverse events were reported by 4% of patients during procedure and by 10% within 30 days. While 38% reported a worse, 62% reported a stable or better global health score, and 54% of patients with worse global health score were treated as salvage therapy patients.

**Conclusion:**

This interim analysis confirms in a prospective analysis the feasibility of the study, with an acceptable toxicity profile. More patients reported a stable or improved HRQOL than deterioration. Deterioration of HRQOL was seen especially in salvage therapy patients.

**Trial Registration:**

NCT03086096.

**Electronic supplementary material:**

The online version of this article (10.1007/s00270-020-02646-8) contains supplementary material, which is available to authorised users.

## Introduction

Transarterial chemoembolisation (TACE) using irinotecan-eluting beads is part of the loco-regional treatments toolbox to control colorectal cancer liver metastases (CRLM) in the 80% of patients that are not eligible for the only proven curative treatment options, surgery and thermal ablation [1, 2]. Clinical trials and case–control studies have evaluated TACE using irinotecan in various settings and indications [3–13]. However, prospective real-world data, from which our understanding of how the treatment is used and how it can be improved, is still missing.

The CIrse REgistry for LifePearl™ microspheres (CIREL) was designed to prospectively capture data of CRLM patients decided to be treated with TACE using irinotecan-eluting LifePearl™ microspheres (LP-irinotecan TACE). The study was carried out by interventional radiologists and supported by multidisciplinary teams, such as oncologists. This interim analysis was performed as a part of the CIREL study protocol in order to assess the feasibility of the study in terms the planned statistical analysis [14] and represents the first insight into the prospective real-life observation of the use of LP-irinotecan TACE from 9 different European teams while also illustrating treatment considerations in terms of bead size and procedural medication and confirming the safety of TACE.

## Methods

For a detailed description of CIREL methodology please refer to Pereira et al. [14].

### Study Design/Setting

Data were supplied by 9 sites in 7 European countries by interventional radiologists who were invited to participate when having experience with the treatment and having performed at least one treatment with LP-irinotecan TACE and either a total of 40 treatments or at least 10 treatments in the last 12 months with any drug-eluting beads.

### Patients

Eligible patients were ≥ 18 years with histologically confirmed colorectal adenocarcinoma with liver-only or liver-dominant metastases, and treatment with LP-irinotecan TACE (LifePearl™ microspheres, Terumo Europe N.V., Leuven, Belgium) decided in a multi-disciplinary tumour board. There were no further inclusion or exclusion criteria. Patients included in this 50-patient interim analysis were enrolled between February 2018 and June 2019. All aspects related to treatments and related to the follow-up protocol were performed at the treating physician’s discretion, including whether treatments were performed under general or local anaesthesia, for which data were not collected.

### Study Objectives and Data Sources

Patients and disease characteristics were collected through an e-CRF using OpenClinica 3. Automatic data checks and verifications were employed as far as possible, and additionally internal data logics were discussed via quarterly remote monitoring.

The primary outcome was the observation of usage of LP-irinotecan TACE by categorising treatment intention as:

1. a first-line therapy for chemo-naïve or patients that have not received systemic chemotherapy after diagnosis of liver metastases,
2. a “consolidation” treatment with or without systemic chemotherapy for patients that have a tumour response or a stable disease on systemic chemotherapy,
3. an intensification treatment with concomitant therapy for patients with progressive disease that have received maximum 2 previous lines of treatment,
4. a salvage therapy for progressive disease patients that have received 3 or more previous lines of chemotherapy,
5. a combination treatment with ablation with curative intent
6. other if none of the previous categories apply.

For details regarding the grouping see supplementary Table 1.

The feasibility of the study was determined by the extent to which planned statistical analyses could be conducted when considering data completion and data distribution of baseline, safety and quality of life data.

Laboratory values were collected 1 to 9 days before LP-irinotecan TACE treatments. Abnormal laboratory values during follow-ups were collected as adverse events. Bead size, the total dose infused, peri-procedural management and technical success (defined as complete delivery of the planned dose or complete stasis), as well as the number of procedures performed per lobe and additional medical and locoregional treatments, were registered and analysed.

All adverse events (AE) were classified according to CTCAE 4.03 (Cancer Institute’s Common Terminology Criteria for Adverse Events 4.03 and 5.0; see supplementary Table 2) [15, 16] and were collected continuously. Health-related quality of life (HRQOL) questionnaires (QLQC30) were collected at 2 different timepoints: at baseline (1–9 days before treatment) and at the first follow-up (4–15 weeks after the last treatment session). HRQOL was assessed according to the EORTC QLQ-C30 Scoring Manual version 3.0 [17–19]. For the global health and the functional score, a high score indicates high health and for the symptom scale a low score indicates few symptoms. Cut-offs for clinically significant improvement (+ 10 for global health, functional score, − 10 for symptom score) or deterioration (− 10 for global health, functional score, + 10 for symptom score) compared to baseline were used [20].

### Bias

Potential bias regarding data correctness and completeness was addressed by a quality assurance system including remote monitoring and data management. No source document verification was performed. Potential selection bias was addressed by contractually agreeing with all sites to present the possibility to participate to all potentially eligible patients. The number for non-inclusion was registered resulting in 56 potentially eligible patients having received LP-irinotecan TACE and 50 patients included in CIREL.

### Statistical Methods

Baseline and treatment characteristics, as well as the primary endpoint of observed usage of LP-irinotecan TACE and secondary endpoints (laboratory data, adverse events and HRQOL), were evaluated using descriptive statistics. For continuous data, median (range) is shown. Categorical data are presented as counts (percentages). Data were plotted using RStudio under R3.6.1.

## Results

### Patients and Disease Characteristics

This interim report includes the prospectively captured real-life use of LP-irinotecan TACE as a treatment for CRLM in the first 50 patients enrolled. Median age was 66 years; 29 patients (58%) were male. Patients and tumour characteristics are summarised in Table 1.

**Table 1:** Tumour and patients’ characteristics

*Primary tumour*, *n* (%)					
Right-sided primary colon cancer (RSP)	10 (20)				
Left-sided primary colon cancer (LSP)	29 (58)				
Rectum cancer (LSP)	11 (22)				
CEA increased	46 (92)				
CA 19.9 increased	25 (50)				
*Primary tumour treatment*, *n* (%)					
Surgery	44 (88)				
Radiochemotherapy	6 (12)				
Systemic chemotherapy	13 (26)				
Targeted therapy	6 (12)				
*Eastern Cooperative Oncology Group (ECOG) performance status*, *n* (%)					
0	36 (72)				
1	11 (22)				
2	3 (6)				
*Primary TNM status*, *n* (%)					
Tis	0	N0	6 (12)	M0	16 (32)
T1	5 (10)	N1a	11 (22)	M1	28 (56)
T2	5 (10)	N1b	8 (16)	Mx	4 (8)
T3	28 (56)	N1c	3 (6)		
T4	10 (20)	N2a	5 (10)		
		N2b	3 (6)		
		Nx	12 (24)		

*Molecular characterisation*					
*RAS, n (%)*					
Yes	17 (34)				
No	24 (48)				
N/A	9 (18)				
*BRAF, n (%)*					
Yes	5 (10)				
No	18 (36)				
N/A	27 (54)				
*Extrahepatic metastases*					
Yes, *n* (%)	16 (32)				
No, *n* (%)	34 (68)				
Median number (larger than 10 mm) (min, max)	2 (1,6)				
*Location*, *n* (%)					
Lymph nodes	5 (31)				
Peritoneum	1 (6)				
Lung	12 (75)				
Bones	1 (6)				
Liver metastases					
*Time since primary cancer diagnosis,* *n (%)*					
Median time for liver metastases since primary tumour diagnosis, years (min, max)	2 (0.3, 9.7)				
Synchronous (< 6 months)	34 (68)				
Metachronous (> 6 months)	16 (32)				
*Location*, *n* (%)					
Whole liver	26 (52)				
Left liver lobe only	7 (14)				
Right liver lobe only	17 (34)				
*Liver tumour burden*, *n* (%)					
< 25%	33 (66)				
25–50%	13 (26)				
> 50%	4 (8)				
*Median size of the two largest lesions, mm (min, max)*					
Lesion 1	54 (10, 132) × 43,5 (10, 127)				
Lesion 2	30 (9, 94) × 25 (8, 87)				
*Number of lesions, n* (%)					
1	8 (16)				
2–3	16 (32)				
4–10	15 (30)				
> 10	11 (22)				
*Previous treatments for liver metastases*, *n* (%)					
Systemic chemotherapy	41 (82)				
1 line	9 (18)				
2 lines	6 (12)				
3 or more lines	26 (52)				
Anti-angiogenic targeted therapy	18 (36)				
Anti-EGFR targeted therapy	10 (20)				
Surgery	10 (20)				
Adjuvant fluoropyrimidine	2 (4)				
Adjuvant oxaliplatin	2 (4)				
Adjuvant irinotecan	2 (4)				
Ablation	5 (10)				
Intra-arterial treatment	6 (12)				

Liver metastases were synchronous in 68% and metachronous in 32% of patients. In total, 52% of those were located in the whole liver, 34%/14% limited to the right/left lobe, respectively. In total, 34% had a proven RAS mutation, 48% RAS wild-type status (18% unknown), 10% a proven BRAF mutation, 36% wild-type status, 54% unknown.

Before receiving LP-irinotecan TACE, 82% (41 patients) had received prior systemic chemotherapy for metastatic disease. In total, 18% (9 patients) were pretreated with one line, 12% (6 patients) with two lines and 52% (26 patients) with 3 or more treatment lines. Of the overall population 36% received anti-angiogenic and 20% anti-EGFR targeted therapy. In 20%, liver metastases had been resected with 4% of the total population having received adjuvant fluoropyrimidines and oxaliplatin and 4% having received adjuvant irinotecan. Prior ablation and intra-arterial liver-directed treatment were seen in 10% and 12% of patients.

### LP-Irinotecan TACE Treatment and Treatment Intention

Information on 129 LP-irinotecan TACE treatment sessions in our 50 patients is shown in Table 2.

**Table 2:** Treatment intentions for LP-irinotecan TACE and treatments’ characteristics

*Treatment intention n* (%)	
First-line treatment	8 (16)
Consolidation or closing treatment with or without systemic therapy	7 (14)
Intensification of treatment with concomitant systemic therapy	10 (20)
Rescue salvage treatment/end-stage treatment in progressive patients pretreated with systemic therapy, with or without concomitant systemic therapy	21 (42)
Combination treatment with ablation with a curative intent	4 (8)
Unilobar treatment *n* (%)	25 (50)
Median number of sessions (min, max)	2 (1, 4)
Right lobe	39
Left lobe	13
*Bilobar treatment n* (%)	25 (50)
Right lobe	45
Left lobe	32
Median number of sessions per patient (min, max)	2,6 (1, 5)
*LP-irinotecan TACE treatments* (*n *= 129)	
Median intended dose per session, mg (min, max)	100 (25, 100)
*Bead size*, *n* (%)	
100 ± 25 µm	111 (86)
200 ± 50 µm	17 (13)
400 ± 50 µm	1 (1)
*Treatment technically successful n* (%)	
Yes	129 (100)
*Technical success related to n* (%)	
Complete stasis	45 (36)
Complete delivery of the dose	82 (64)

In total, 50% of patients received treatments in only one liver lobe (unilobar), with a median number of 2 sessions per patient (78% right lobe, 22% left lobe). The median number of treatments where both lobes were targeted alternatingly (bilobar) per patient was 2.6 sessions. The median intended dose per session was 100 mg (80% of patients) using a bead size of 100 µm (86%). Treatments were considered technically successful in 100% of cases, showing either a complete stasis in 36% or a complete delivery of dose in 64%.

For most patients, intention of LP-irinotecan TACE was *salvage therapy* (42%; 21 patients), meaning the patients had progressive disease and received three or more lines of chemotherapy before. LP- irinotecan TACE treatment was intended in 20% (10 patients) as an *intensification treatment with concomitant systemic therapy* for patients with progressive disease but maximum two previous lines of chemotherapy and 14% (7 patients) as a *consolidation treatment* with or without systemic chemotherapy for patients with stable disease. Only 16% and 8% (8 and 4 patients, respectively) of treatments were classified as *first line* for chemo-naive patients or patients that have not received prior systemic chemotherapy for the liver metastases and *combination treatment with ablation with curative intent*, respectively. Treatment intentions were distributed evenly across high-enrolling sites (supplementary Table 3).

**Fig. 1:**
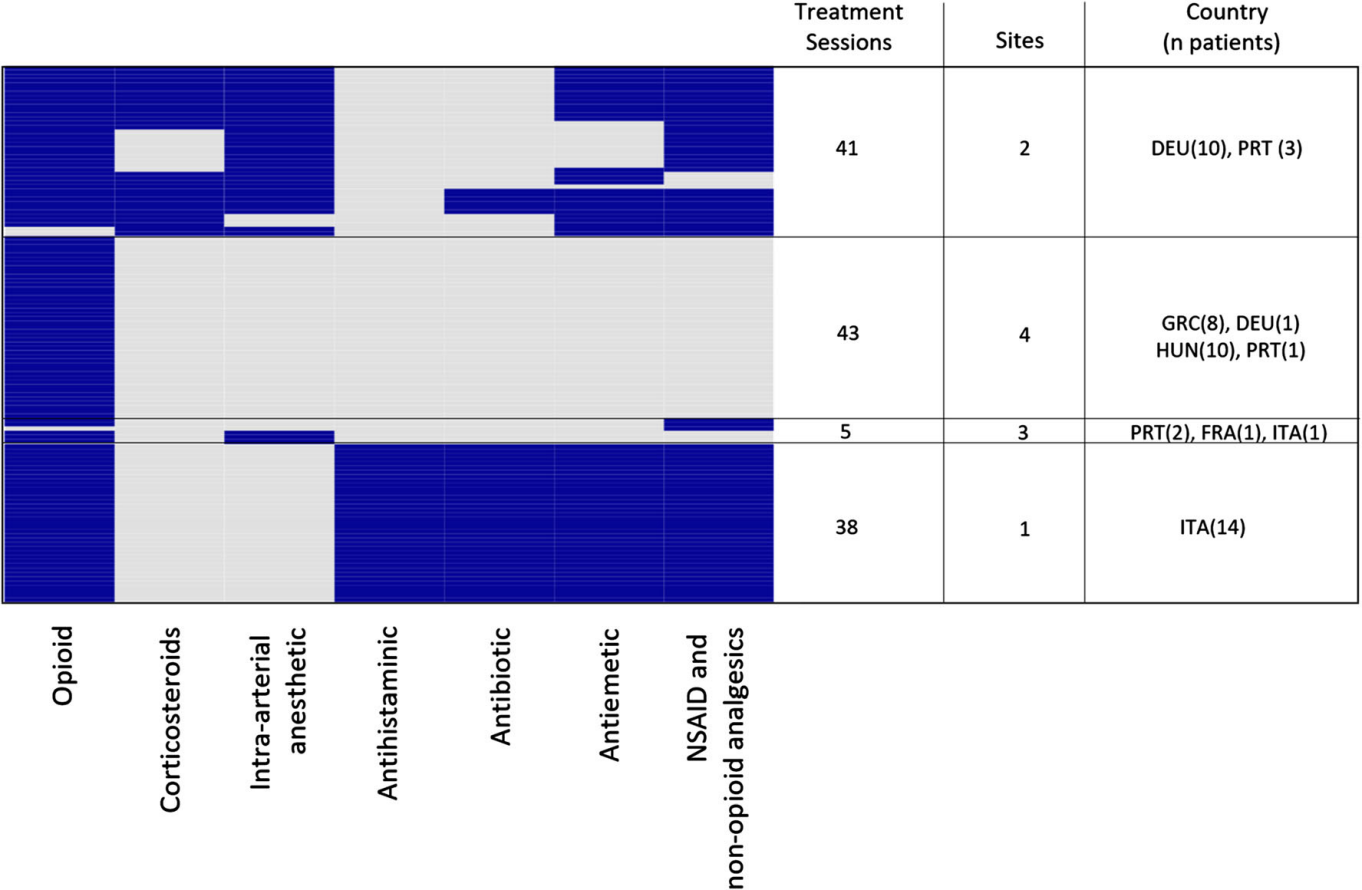
Procedural medications used during LP-irinotecan TACE treatments. Depicts a heatmap of all medications used during each LP-irinotecan TACE treatment session (*n *= 127) divided into 4 groups based on similar medications used and a table listing the number of treatment sessions, different sites, as well as country (number of patients) per group. Each row represents one treatment, and each column represents the used class of medication. Coloured fields indicate that during the treatment the respective medication was used. 1 patient (in 2 treatments) where epidural anaesthesia was used in the absence of any other procedural medication is not shown. The heatmap was generated using the R heatmap function with the default clustering algorithm. Dendrograms for the clustering algorithm are not shown

When analysing peri-procedural medications, we observed different treatment strategies in different sites as seen in Fig. 1. While opioids were used in almost all sessions, some patients received additional medication such as local anaesthesia, additional non-opioid pain- or anti-inflammatory medication, anti-histamine, antibiotics and antiemetics.

### Safety and Toxicity

Abnormal laboratory values of grade 3 or 4 were observed 7 times in 4 patients before the first treatment and 11 times in 5 patients before subsequent LP-irinotecan TACE treatments (Table 3). Haematological, renal and hepatic toxicity was only observed by single abnormal laboratory values of grade 3 and 4 in individual patients with no increase compared to abnormal laboratory values of grade 3 and 4 before the first treatment session.

**Table 3:** Pathological laboratory values before and after LP-irinotecan TACE treatments

CTCAE 4.03. grade	Grade 1	Grade 2	Grade 3	Grade 4
*Max 7* *days before first LP-irinotecan TACE treatment*				
Serum creatinine increased	2	0	0	2
Bilirubin increased	5	2	0	0
SGPT increased	12	2	0	0
SGOT increased	15	1	0	0
Albumin decreased	0	1	0	0
LDH increased	20	N/A	N/A	N/A
Alkaline phosphatase increased	20	4	2	0
Neutrophils decreased	1	0	0	1
Platelets decreased	8	0	0	0
Lymphocytes decreased	7	4	1	1
*1–9* *days before consecutive treatments*				
Serum creatinine increased	4	0	0	0
Bilirubin increased	6	3	0	0
SGPT increased	26	5	1	0
SGOT increased	28	2	0	0
Albumin decreased	0	3	0	0
LDH increased	27	N/A	N/A	N/A
Alkaline phosphatase increased	44	10	5	0
Neutrophils decreased	2	0	0	1
Platelets decreased	5	0	0	0
Lymphocytes decreased	21	5	2	2

One session from one patient was removed because the date of blood sampling was out of time range (48 days)

Adverse events (AE) are summarised in Table 4. In total, 33 peri-interventional AEs were reported, with 26% of patients having experienced at least one AE. Most notably, patients were experiencing grade 1 and grade 2 post-embolisation syndrome, which is defined as pain, fever or nausea/vomiting, with grade 1 pain being reported most frequently.

**Table 4:** Adverse events experienced peri-interventionally and within the first 30 days after LP-irinotecan TACE treatment

	Total AEs	Patients with at least one AE (%)	Total serious AEs (grade 3 + 4)	Patients with at least one serious AE (%) (grade 3 + 4)
Peri-interventional	33	13 (26)	2	2 (4)
Within the first 30 days after treatment	24	10 (20)	7	5 (10)

	Grade 1	Grade 2	Grade 3	Grade 4
*Peri-interventional*				
Infusion-related reaction			1	
Pain	23	1		
Vomiting	2			
Hypertension			1	
Nausea	4			
Dislocation of one coil during coiling of A. cystica		1		

	Grade 1	Grade 2	Grade 3	Grade 4
*Within the first 30* *days after treatment*				
Pain	2	2		
Fever	1	1		
Nausea	1	1		
Vomiting		1		
Diarrhoea		1		
Alopecia	1			
Hepatic failure			1	
Cholecystitis		1		
Sepsis				1
Colonic obstruction				1
Abdominal abscess		1		
Liver abscess			1	
Ascites		1		
Asthenia		1		
Platelet count decreased	1	1		
Blood bilirubin increase			1	
Infection, CRP increasing			1	
Renal failure + hyperkalemia			1	

Within 30 days following the last treatment, 20% (10 patients) experienced at least one AE. Grade 3 and 4 AEs were reported for 10% (5 patients). One patient experienced a grade 4 AE (colonic obstruction and sepsis), as well as a grade 3 AE (hepatic failure and blood bilirubin increase). Another patient experienced a grade 3 AE (renal failure and hyperkalemia), and one patient a grade 3 AE (infection and CPR increase) and one last patient experienced a grade 3 AE(abscess). No adverse event resulting in mortality was reported in the 30 days after the last treatment.

### Health-Related Quality of Life Analysis

**Fig. 2:**
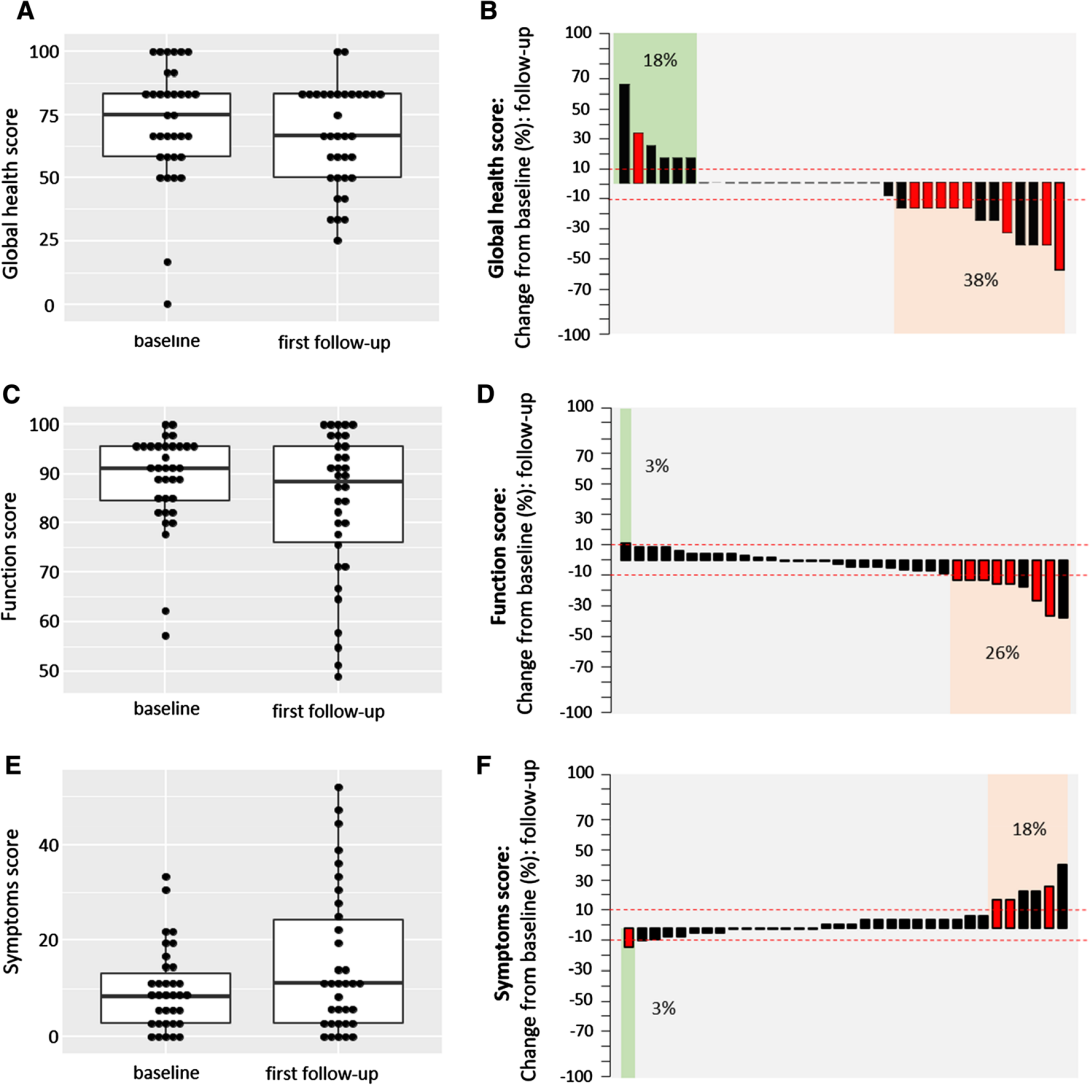
Health-related quality of life according to EORTC-QLQ 30. Shows HRQOL score of 34 patients collected at baseline (before the first treatment) and at the first follow-up (4–15 weeks later) by analysing global health (**a**, **b**), function (**c**, **d**) and symptoms (**e**, **f**) score according to EORTC-QLQ30 version 3.0. For the global health and the function score, a high score indicates high health and for the symptom scale a low score indicates few symptoms. For general comparisons between baseline and the first follow-up, boxplots were used (**a**, **c**, **e**). The difference between baseline and first follow-up was plotted for individual patients using a waterfall diagram. Cut-offs (dashed line) for clinically significant improvement were set at + 10 for global health, functional score and − 10 for symptom score and at − 10 for global health, functional score and + 10 for symptom score for deterioration. Red bars indicate patients with treatment intention: salvage therapy (see supplementary Table 1)

Median HRQOL deteriorated over time, as both—median global health and median functional—scores decreased and the median symptom score increased (Fig. 2). Figure 2a, c, e shows the difference in median HRQOL for 34 patients for the first follow-up compared to the baseline scores of the same patients. Median global health, functional and symptom score before the first LP-irinotecan TACE treatment was 75.0, 91.1 and 8.3, respectively, and 66.7, 88.5 and 11.1, at the first follow-up. When looking at the patients individually, we see that for the majority of patients the global health, functional and symptom scores remained similar or improved (Fig. 2b, d, f).

In total, 62% of patients reported that the global health status stayed the same or improved (18%) and 38% of patients showed a decrease of global health. For the functional score, 74% of patients reported a stable or increased (3%) score and 26% of patients reported a worsening of the functional score. For the symptom score 82% reported stable or improved (3%) score and 18% reported worsening of the symptom score. When looking at patients with deterioration in global health, functionality and symptoms, we observed that 54%, 77% and 50%, respectively, were salvage therapy patients (see Fig. 2b, d, f, red bars).

## Discussion

While TACE is a first-line treatment option in intermediate hepatocellular carcinoma (HCC) [21, 22], CRLM TACE with irinotecan is a niche treatment with many competing interventional treatment alternatives such as thermal ablation, intra-arterial chemotherapy [23] and radioembolisation [1]. Additionally, systemic treatments including chemotherapy and targeted therapies, usually anti-VEGF or anti-EGFR antibodies, are used throughout the entire cancer continuum of care [1] and are the subject of many currently running trials [24–26]. Due to this competition of treatments experienced in daily clinical practice and considering that interventional radiologists are not always part of multidisciplinary tumour boards, difficulties in centre and patient enrolment were encountered in CIREL. Due to this lower than expected enrolment, the CIREL Steering Committee decided to end patient enrolment after 30 months instead of the initially planned 36 months and to increase the follow-up period to allow capturing of high-quality data and estimating meaningful survival data [14].

Despite these obstacles, CIREL will be the largest multicentre, prospective observational study on the real-life use of TACE with irinotecan using LifePearl™ microspheres in Europe and the first prospective study to comprehensively categorise treatment intention for irinotecan TACE in CRLM.

For this, it was considered whether patients had received previous systemic therapy, how many lines thereof, and at which stage of the cancer continuum of care the treatment was performed (supplementary Table 1). An additional group was LP-irinotecan TACE as a combination treatment with ablation with a curative intent. Typically, TACE with irinotecan is mainly used as a salvage therapy, with no consistent definitions of the number of previous lines of chemotherapy across already published studies [27–30] . Furthermore, evaluation of treatment intention is lacking. With LP-irinotecan TACE being used in 42% of patients as a salvage therapy, CIREL has not only prospectively evaluated and precisely described usage of LP-irinotecan TACE as a salvage treatment, but also differentiated between other treatment intentions, e.g. usage as a consolidation or intensification therapy. In this context, the results of CIREL will also improve our understanding of LP-irinotecan TACE used in clinical settings which are less commonly studied such as in chemotherapy-naive patients, only reported on in a few studies [10, 31]. With a bigger sample size and in relation to these categories, effectiveness outcomes like overall survival and (hepatic) progression-free survival will be reported in the final analysis.

The vast majority (86%) of treatments was performed using 100-µm beads. While available bead sizes range from small (70–150 µm) to very large (500–700 µm), smaller beads (70–150 or 100–300 µm) [32, 33] are recommended based on studies, suggesting that best clinical results can be achieved with small beads [34–37]. However, whether 70–150-µm beads lead to better results is still a matter of debate [38] and a recent study by Boeken et al. showed no significant difference regarding patient outcome when using different bead sizes [39].

In contrast to doxycycline-TACE in intermediate HCC, there is no standardisation of peri-procedural management for irinotecan TACE. As expected, this has resulted in vast differences in procedural medications being reported in CIREL, which has been already reported in the literature [40]. In response to the need for treatment standardisation, Iezzi et al. recently published recommendations, suggesting the continuous infusion of opioid and non-steroidal anti-inflammatory medication to reduce peri-procedural pain. At the physician’s discretion they suggest the prophylactic administration of antibiotics before and after treatment as well as the intra-arterial lidocaine administration directly prior to bead injection [33].

The appropriate peri-procedural management is important to control treatment-associated adverse events such as the commonly experienced grade 1 or 2 post-embolisation syndrome (PES). PES (grade 1) is also the most common adverse events (AE) by far in this interim analysis. While the number of reported grade 1 and grade 2 AEs varied highly, most studies reported none [36, 41, 42]] or below 10% [10, 27, 31, 43] adverse events of grade 3 and 4. This is in line with what was observed in CIREL with 10% of grade 3 reported adverse events and only 4% of grade 4 in this interim analysis. However, correlating different peri-procedural medications and treatment strategies with adverse events is beyond the scope of this interim analysis.

Another important aspect of palliative treatment is the maintenance of health-related quality of life (HRQOL), yet there are only few studies dedicated to studying HRQOL in TACE in CRLM [44–47]. Our data suggest improvement or maintenance of global health in 62%, of functionality in 74% and of symptom score in 82% of patients. Deterioration in HRQOL global health score was observed especially for salvage therapy patients. While reporting maintenance or improvement of HRQOL, most studies have a limited number of participants [46, 47] or are not using patient-reported performance scales [45] and there is currently no comparable study describing the effect on irinotecan TACE on global health and functionality score. Previous findings using the Edmonton SAS questionnaire which correlates with the symptom score of the EORTC QLQ 30 in a comparable cohort to CIREL report improvement in 91% of patients [44] which is in line with our results regarding the symptom score. Using the comprehensive patient-reported EORTC QLQ 30-questionnaire scoring system, in the final analysis CIREL will give relevant insights into HRQOL for patients with different treatment intentions and into whether a gain in overall survival can be observed despite a deterioration of global health in salvage therapy patients.

By collecting data from different sites and countries across Europe, we could already show notable preliminary data regarding safety, bead size and periprocedural medications. In the final analysis, CIREL will elaborate on heterogeneity and homogeneity of compliance to guidelines and suggestions regarding technical aspects of treatment administration.

### Study Limitation

The data for this interim analysis are from 50 patients from 9 centres in 7 European countries. The final data set will include patients from 25 different centres in 12 different countries. Therefore, conclusions could be subject to change as standard practices in different centres and countries could differ. Additionally, as analysing all effectiveness outcomes is beyond the scope of this interim analysis considering that follow-up data collection is ongoing and the results cannot yet be analysed in conjunction with outcomes such as overall survival, hepatic-free survival.

## Conclusion

This interim analysis illustrates that in CIREL, LP-irinotecan TACE was mainly used as salvage or intensification therapy with an acceptable toxicity profile. HRQOL of the global health score deteriorated in more patients than the functional and symptom score, for both of which over 70% of patients reported stable or improved scores at the first follow-up compared to baseline. Additionally, this interim analysis illustrates the feasibility of categorising the use of LP-irinotecan TACE and studying quality of life in terms of data completeness and distribution. Therefore, the final results of CIREL will be able to provide prospective and meaningful data for the use and safety of TACE using irinotecan in different settings across multiple sites in Europe and analyse HRQOL using the comprehensive EORTC questionnaire.

## Electronic supplementary material

Below is the link to the electronic supplementary material.

Supplementary material 1 (DOCX 24 kb)

Supplementary material 2 (DOCX 25 kb)

Supplementary material 3 (DOCX 25 kb)
