# Supplementary material for: The CIREL Cohort: A Prospective Controlled Registry Studying the Real-Life Use of Irinotecan-Loaded Chemoembolisation in Colorectal Cancer Liver Metastases: Interim Analysis
Source: Cardiovasc Intervent Radiol. 2020 Sep 24;44(1):50–62. doi: 10.1007/s00270-020-02646-8 (PMC7728640; doi:10.1007/s00270-020-02646-8)
Supplement: Supplementary file 1 — Supplementary material 1 (DOCX 24 kb) [file 270_2020_2646_MOESM1_ESM.docx]

Supplementary table 1: Categories used to describe treatment intention with LP-irinotecan TACE

| **Category** | **Patient characteristics** |
| --- | --- |
| 1. First-line | Chemo-naive (synchronous)  No systemic chemotherapy after LM diagnosis (metachronous) |
| 2. Consolidation therapy (with/without systemic chemotherapy) | Stable disease  Previous systemic chemotherapy |
| 3. Intensification of treatment with concomitant therapy | Progressive disease  Previous systemic chemotherapy  Max 2 lines of systemic chemotherapy |
| 4. Salve treatment in progressive patients pretreated with systemic chemotherapy | Progressive disease  Min 3 lines of systemic chemotherapy |
| 5. Combination treatment with ablation with a curative intent | Ablation performed after or during LP-IRI chemoembolisation |
| 6. Other |  |
