# Supplementary material for: The CIREL Cohort: A Prospective Controlled Registry Studying the Real-Life Use of Irinotecan-Loaded Chemoembolisation in Colorectal Cancer Liver Metastases: Interim Analysis
Source: Cardiovasc Intervent Radiol. 2020 Sep 24;44(1):50–62. doi: 10.1007/s00270-020-02646-8 (PMC7728640; doi:10.1007/s00270-020-02646-8)
Supplement: Supplementary file 2 — Supplementary material 2 (DOCX 25 kb) [file 270_2020_2646_MOESM2_ESM.docx]

Supplementary table 2: Abnormal laboratory values graded according to CTCAE 4.03 and 5.0

| **CTCAE 4.03** | | | | |
| --- | --- | --- | --- | --- |
|  | Grade 1 | Grade 2 | Grade 3 | Grade 4 |
| Serum creatinine increased | >1 - 1.5 x baseline; >ULN - 1.5 x ULN | >1.5 - 3.0 x baseline; >1.5 - 3.0 x ULN | >3.0 baseline; >3.0 - 6.0 x ULN | >6.0 x ULN |
| Bilirubin increased | >ULN >ULN - 1.5 x ULN | >1.5 - 3.0 x ULN | >3.0 - 10.0 x ULN | >10.0 x ULN |
| Alt SGT increased | >ULN - 3.0 x ULN | >3.0 - 5.0 x ULN | >5.0 - 20.0 x ULN | >20.0 x ULN |
| AST SGOT increased | >ULN - 3.0 x ULN | >3.0 - 5.0 x ULN | >5.0 - 20.0 x ULN | >20.0 x ULN |
| Albumin decreased | <LLN - 3 g/dL; <LLN - 30 g/L | <3 - 2 g/dL; <30 - 20 g/L | <2 g/dL; <20 g/L | Life-threatening consequences; urgent intervention indicated |
| Alkaline phosphatase increased | >ULN - 2.5 x ULN | >2.5 - 5.0 x ULN | >5.0 - 20.0 x ULN | >20.0 x ULN |
| Neutrophils decreased | <LLN - 1500/mm3; <LLN - 1.5 x 10e9 /L | <1500 - 1000/mm3; <1.5 - 1.0 x 10e9 /L | <1.0 - 0.5 x 10e9 /L | <500/mm3; <0.5 x 10e9 /L |
| Platelets decreased | <LLN - 75,000/mm3; <LLN - 75.0 x 10e9 /L | <75,000 - 50,000/mm3; <75.0 - 50.0 x 10e9 /L | <50.0 - 25.0 x 10e9 /L | <25,000/mm3; <25.0 x 10e9 /L |
| Lymphocytes decreased | <LLN - 800/mm3; <LLN - 0.8 x 10e9/L | <800 - 500/mm3; <0.8 - 0.5 x 10e9 /L | <0.5 - 0.2 x 10e9 /L | <200/mm3; <0.2 x 10e9 /L |
| **CTCAE 5.0** | | | | |
| LDH increased | >ULN |  |  |  |
