# Supplementary material for: The CIREL Cohort: A Prospective Controlled Registry Studying the Real-Life Use of Irinotecan-Loaded Chemoembolisation in Colorectal Cancer Liver Metastases: Interim Analysis
Source: Cardiovasc Intervent Radiol. 2020 Sep 24;44(1):50–62. doi: 10.1007/s00270-020-02646-8 (PMC7728640; doi:10.1007/s00270-020-02646-8)
Supplement: Supplementary file 3 — Supplementary material 3 (DOCX 25 kb) [file 270_2020_2646_MOESM3_ESM.docx]

Supplementary table 3: Distribution of treatment intention from high-enrolling centres (n>=5).

| **Treatment intention** | **site 1 – ITA**  **n=14** | **site 2- GER**  **n=10** | **site 3 -HUN**  **n=9** | **site 4 -GRC**  **n=8** | **site 5 – PRT**  **n=5** |
| --- | --- | --- | --- | --- | --- |
| 1. First line | 1 (7%) |  | 3 (33%) | 1 (12%) | 2 (40%) |
| 2. Consolidation therapy (with/without systemic chemotherapy) | 2 (14%) | 1 (10%) | 1 (11%) | 1 (13%) | 2 (40%) |
| 3. Intensification of treatment with concomitant therapy | 3 (21%) | 3 (30%) | 2 (22%) | 1 (13%) |  |
| 4. Salve treatment in progressive patients pretreated with systemic chemotherapy | 6 (43%) | 5 (50%) | 3 (33%) | 4 (50%) | 1 (20%) |
| 5. Combination treatment with ablation with a curative intent | 2 (14%) | 1 (10%) |  | 1 (13%) |  |
